# Withholding and withdrawal of life-sustaining treatments in intensive care units in Lebanon: a cross-sectional survey of intensivists and interviews of professional societies, legal and religious leaders

**DOI:** 10.1186/s12910-020-00525-y

**Published:** 2020-08-28

**Authors:** Rita El Jawiche, Souheil Hallit, Lubna Tarabey, Fadi Abou-Mrad

**Affiliations:** 1 Anesthesia Department, Bahman Hospital, Haret Hreik, near Masjed El Hassanein, Beirut, Lebanon; 2 Faculty of Medicine and Medical Sciences, Holy Spirit University of Kaslik (USEK), Jounieh, Lebanon; 3 INSPECT-LB: Institut National de Santé Publique, Epidemiologie Clinique et Toxicologie- Liban, Beirut, Lebanon; 4 Institute of Social Sciences and Medical School, Lebanese University, Hadath, Lebanon; 5 Neurology Division and Memory Clinic, Saint Charles Hospital, Baabda, Lebanon; 6 Division of Medical Ethics & Forensic Medicine, Lebanese University, Hadath, Lebanon

**Keywords:** Withholding, Withdrawal, Life-sustaining treatments, ICU, Lebanon, Middle East

## Abstract

**Background:**

Little is known about the attitudes and practices of intensivists working in Lebanon regarding withholding and withdrawing life-sustaining treatments (LSTs). The objectives of the study were to assess the points of view and practices of intensivists in Lebanon along with the opinions of medical, legal and religious leaders regarding withholding withdrawal of life-sustaining treatments in Lebanese intensive care units (ICU).

**Methods:**

A web-based survey was conducted among intensivists working in Lebanese adult ICUs. Interviews were also done with Lebanese medical, legal and religious leaders.

**Results:**

Of the 229 survey recipients, 83 intensivists completed it, i.e. a response rate of (36.3%). Most respondents were between 30 and 49 years old (72%), Catholic Christians (60%), anesthesiologists (63%), working in Beirut (47%). Ninety-two percent of them were familiar with the withholding and withdrawal concepts and 80% applied them. Poor prognosis of the acute and chronic disease and futile therapy were the main reasons to consider withholding and withdrawal of treatments. Ninety-five percent of intensivists agreed with the “Principle of Double Effect” (i.e. adding analgesia and or sedation to patients after the withholding/withdrawal decisions in order to prevent their suffering and allow their comfort, even though it might hasten the dying process). The main withheld therapies were vasopressors, respiratory assistance and CPR. Most of the respondents reported the decision was often to always multidisciplinary (92%), involving the family (68%), and the patient (65%), or his advance directives (77%) or his surrogate (81%) and the nurses (78%). The interviewees agreed there was a law governing withholding and withdrawal decisions/practices in Lebanon. Christians and Muslim Sunni leaders declared accepting those practices (withholding or withdrawing LSTs from patients when appropriate).

**Conclusion:**

Withholding and withdrawal of LSTs in the ICU are known concepts among intensivists working in Lebanon and are being practiced. Our results could be used to inform and optimize therapeutic limitation in ICUs in the country.

## Background

During the past 50 years, life-sustaining treatments have been introduced into clinical practice (e.g., mechanical ventilation, dialysis, feeding tubes, etc.). These treatments sustain and prolong lives of patients with conditions that otherwise would be fatal. Some patients, despite the availability of these treatments, choose to forgo them or request their withdrawal after they are initiated as the treatments are inconsistent with their healthcare values and goals. Such decisions may raise moral or ethical concerns about the right for a patient to die in dignity while receiving non-beneficial treatments [1–6]. The two main aspects of this approach are the withholding and withdrawal of life-sustaining treatments; “*Withholding*” being defined as the decision not to start or increase a life-sustaining intervention, and “*Withdrawal”* as the decision to actively stop a life-sustaining intervention presently being given. Currently, these decisions are being extensively applied in ICUs. The associated ethical issues and emotional burden for the patients, their families and the medical ICU staff, encourages the regular evaluation of these decisions’ process, in our personal and institutional medical practice [7–10]. Scientific societies over the world have endorsed guidelines and promoted recommendations to implement these concepts in the ICU [2, 4, 7, 11–15]. In some countries, they are even controlled by laws [13, 16, 17]. Clinical studies have been published, especially from North America [11, 18, 19] and Europe [2, 7, 16, 17, 20–26] describing and assessing those practices. However, there are very few from Middle Eastern Arabic countries, where ethical values, medical resources, and financial limitations are different from those in Western countries, and where the religious and socio-cultural influences have a major impact [10, 27–30].

Lebanon is a Middle Eastern Arab country where many religions and confessions coexist, along with oriental and occidental influences. In the past 20 y, two studies only were reported in the country regarding withholding and withdrawal of life-sustaining treatments practices. Considering all possible religious and socio-cultural influences over this ethical subject, we aimed to assess the actual facts surrounding these decisions in Lebanese ICUs. In this study, we undertook an e-survey to determine the views of intensivists in Lebanon in terms of acceptance of these concepts and their application, the reasons to consider withholding and withdrawal, the therapies withheld and/or withdrawn, the decision-making process and the legal awareness of the intensivists regarding these practices. We also conducted interviews with opinion leaders in the country (medical, legal and major religious thought leaders) to catalogue their position about this subject.

## Methods

### Study participation

A web-based survey of intensivists working in adult ICUs and interviews of medical professional, legal and religious leaders in Lebanon, were conducted between September 2017 and February 2018. The questionnaire was aimed at all intensivists (i.e. physicians who manage patients in ICUs) who practice in Lebanon. We defined intensivists as specialized intensive care physicians who either have their main workplace in the ICU or elsewhere and take care of ICU patients on shifts. The predominant sampling frame used was lists of intensivists, registered in this specialty, obtained from national critical care societies and networks (LSA: Lebanese Society of Anesthesiologists; LSCCM: Lebanese Society of Critical Care Medicine). When such lists were incomplete, regional and personal snowball sampling was used as a supplementary method. Notably, anesthesiologists could be registered in both societies if they work in ICU too. However, the questionnaire could be answered once per doctor. Intensivists practicing in an adult ICU, in Lebanon, for a full or limited time of their work schedule, were included. The e-survey was sent to the whole list of email addresses provided by the critical care societies (LSA and LSCCM).

The interviews were conducted with the head of the Lebanese Order of Physicians (LOP) in Beirut, the heads of the LSA, and the LSCCM, and the vice-president of the Lebanese National Consultative Committee on Ethics (LNCCE), a representative of the medical legal opinion in Lebanon (the lawyer of LOP), and representatives of the main religious confessions in the country (Christian Catholic and Orthodox, Muslim Sunni and Shia, and Druze). No biomedical ethicists were interviewed.

### Questionnaire and interviews development

The questionnaire (Additional file 1) and interviews items were generated after an extant literature review of published studies and surveys, exploring the international and the Middle East literature, which revealed to be poor on the subject. Questions and response categories were formulated, assessed, refined or rejected in close cooperation between the authors. A pilot study was conducted on 10 intensivists to make sure that all questions were clear and understandable before starting the data collection.

The e-survey was available in English and French, the two main scientific languages used in the country. It was sent in November 2017. Three reminders were then sent at 2, 4 and 6-week intervals. In parallel, the interviews were progressively conducted during the study period. The interviews were conducted either in English, or French or Arabic, depending on the first language of the interviewee. The survey consisted of 31 close-ended questions (27 single choice questions, 3 multiple choice questions and 1 Boolean questions) and 2 complementary open ended questions, globally divided in 5 sections: (1) information about the responder, his/her clinical practice, and institution, (2) reasons to consider withholding/withdrawal life-sustaining therapy in the ICU, (3) decision-making process (4) practices of withholding/withdrawal and (5) documentation and legal aspects. Definitions were included to help intensivists answer the questions (see Additional file 1). Responses were ranked on a 5-point Likert scale where relevant. The interviews followed the same outline, stressing on the medical, legal, ethical or religious aspects of the subject respectively.

### Statistical analysis

We reported the numbers and percentages of responders providing a given answer among the total sample. For multiple choice questions, we calculated and reported the numbers and percentages of each answer among total respondents. For questions with answers following a Likert scale, the five response categories were reduced to four groups: “Always”, “Often” and “Sometimes” remained as one category each, “Rarely” and “Never” were collapsed into one category for convenience. The original results can be found as additional table (Additional file 2), whereas the merged results are the one used and available within the paper. The SPSS version 23 was used for all statistical analysis.

## Results

### Questionnaire survey

We could track a total of 229 intensivists (134 anesthesiologists/95 non-anesthesiologists); 83 participants completed the full survey. Thus, the response rate was 36.2% (83/229).

#### Demographics

The majority of respondents were between ages 30 and 39 years (45%), men (61%) and Christian Catholic (60%). About half (47%) work in Beirut Table 1 shows the characteristics of the respondents and the centers where they work.

**Table 1:** Demographics of respondents and their centers (*N* = 83 participants)

Characteristics	n*	(%)**
***Gender***		
Male	51	61
Female	32	39
***Age, years***		
< 30	3	4
30–39	37	45
40–49	22	27
50–59	16	19
≥ 60	5	6
***Religion***		
Christian	57	68
Muslim	18	22
Druze	1	1
Atheist	7	8
***Type of Hospital***		
University	49	59
Non-university	13	16
Private	36	43
Public	2	2
***Size of hospital, No. of beds***		
< 200	34	41
200–399	39	47
≥ 400	10	12
***Location of hospital***		
Beirut	39	47
Mount Lebanon	23	28
North Lebanon	11	13
South Lebanon	6	7
Bekaa	3	4
***Numbers of years of practice, years***		
< 5	21	25
5–9	26	31
10–19	18	22
≥ 20	18	22
***Specialty***		
CCM & Anesthesia	52	63
CCM & Pulmonary Medicine	28	34
CCM & Other	3	3
***Fellowship Program Abroad***		
Yes	70	84
No	13	16
***Average working time in ICU***		
Permanently	11	13
≥ 50%	30	36
< 50%	42	51
***Type of ICU***		
Mixed Adult ICU	51	61
Surgical Adult ICU	18	22
Medical Adult ICU	12	14
***Size of ICU, No. of beds***		
< 5	5	6
5–9	59	71
≥ 10	18	22

(*Abbreviation*: *CCM* Critical Care Medicine, *ICU* Intensive Care Unit)

(*Different *n* due to missing data)

(**Percentages may not total 100 because of rounding)

#### Withholding/withdrawal of life-sustaining therapies in the ICU

##### Reasons

**Fig. 1:**
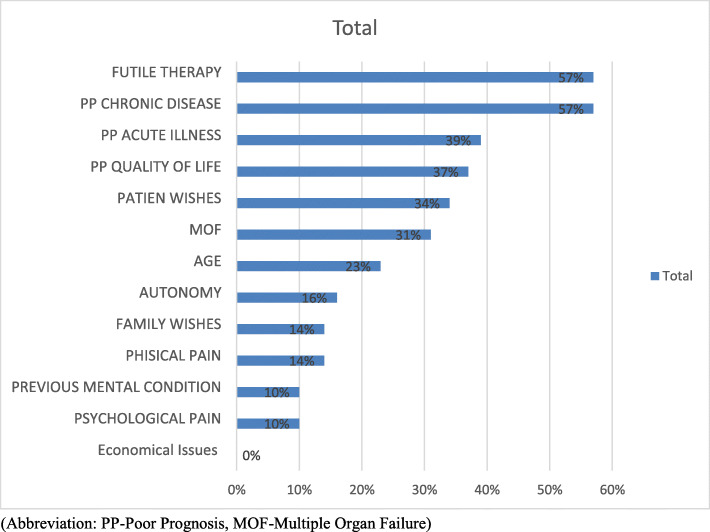
Intensivists’ reasons for considering withholding/withdrawal of life-sustaining therapies in the ICU

The majority of the intensivists was familiar with the withholding/withdrawal concepts from the ethical aspect (92%), 80% of them reported carrying out requests to withhold or withdraw life-sustaining treatments (LST) in their ICU. The majority perceived a difference between withholding and withdrawal of therapy, with withdrawal being more difficult to practice (75%). In the open-ended question (4.c.) exploring the reason for this difficulty, the main answers were that withdrawal is a more active action than withholding. It’s an active way of stopping treatments that will definitely precipitate death, thus, it could be seen as a form of euthanasia, it can cause medico-legal issues, and it might be negatively perceived by the patient’s family, the society and religion. The reasons that caused consideration about withholding/withdrawal therapies are shown in Fig. 1. The top three reasons were “Poor prognosis for the underlying chronic disease” equally with “Futile therapy”, and “Poor prognosis for the acute illness”. The economical and the resources issues were not mentioned at all (0%).

##### Decision-making process

**Fig. 2:**
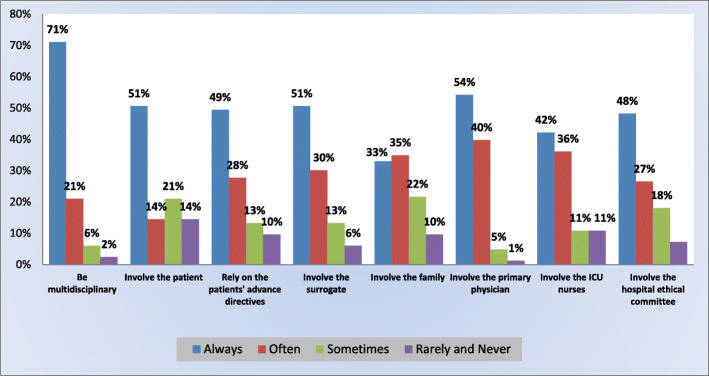
Intensivists’ beliefs about the decision making process regarding the withholding/withdrawal of life-sustaining treatments in ICU: who should be involved?

Nineteen percent of the respondents reported having in their ICU, standardized protocols for the withholding/withdrawal decision-making. Figure 2 presents the views of intensivists on different collaborative aspects of the decision-making process. The majority (92%) of the intensivists agreed that withholding/withdrawal decisions should be multidisciplinary often to always. Many respondents agreed that the patient should often/always take part in this personal decision; 65% would often/always involve the patient, if competent, 77% would often/always rely on the patient’s advance directives, 81% would often/always involve his surrogate. The latter would be much more involved than the family and relatives (68%). Nearly all intensivists (94%) found that the primary treating physician should often/always be integrated in the decision. Moreover, 78% of intensivists would ask for the ICU nurses’ opinion most of the time (i.e. often/always). More than half of the respondents (75%) would frequently include the hospital ethical committee in those decisions (often to always). Almost all intensivists (98%) consented to provide most of the time (i.e. often/always) complete, clear information about the patient’s medical status to the family/relatives or surrogate prior to the decision-making process, no one would rarely/never do that.

##### Practices

**Fig. 3:**
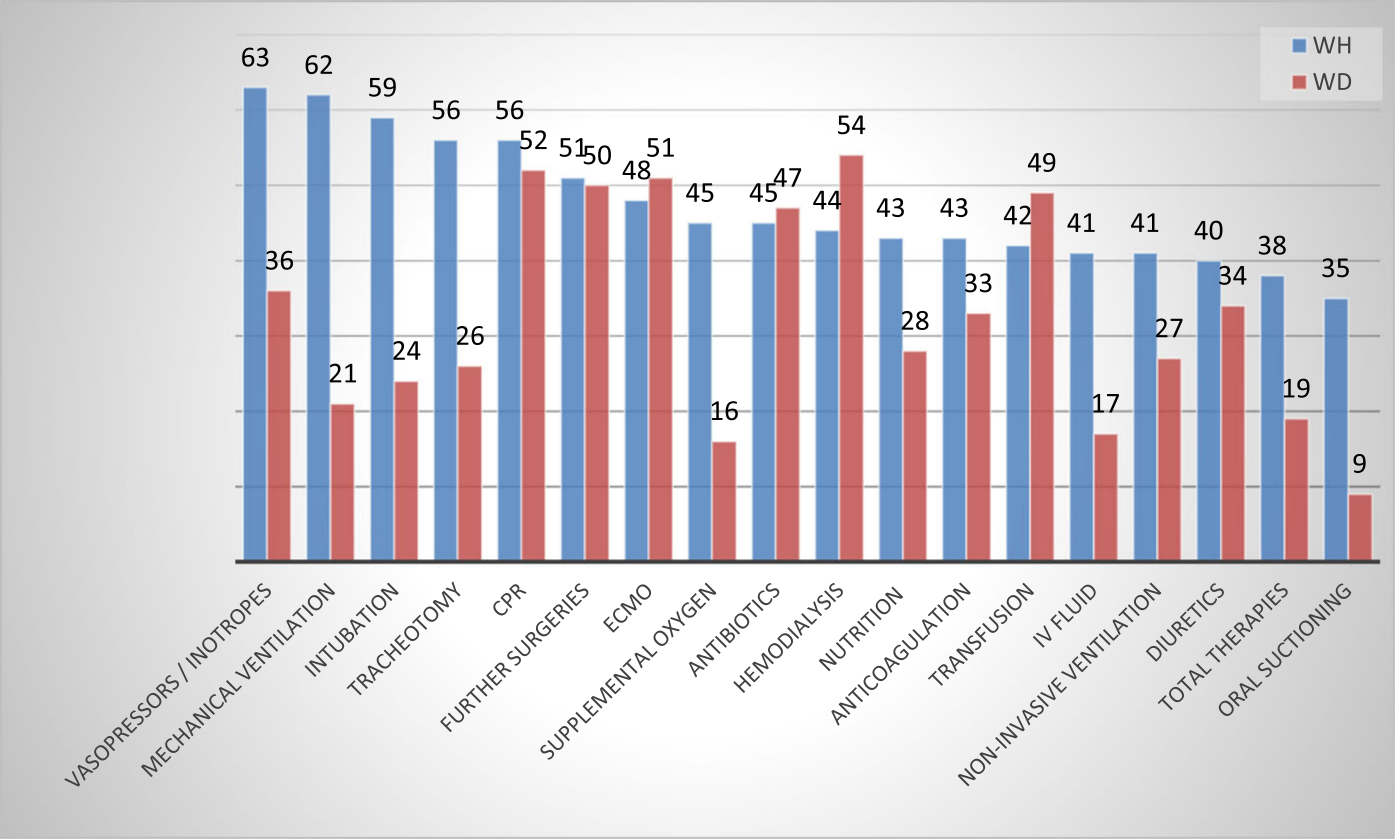
Treatments that could be ethically withheld and/or withdrawn according to the intensivists’ opinion

The majority of respondents reported that LST, including Cardio-Pulmonary Resuscitation (CPR), vasopressors/inotropes, hemodialysis and Extracorporal Membrane Oxygenation (ECMO), transfusion, antibiotic and anticoagulation therapies could usually be withheld and withdrawn. As for, nutrition (enteral and parenteral), supplemental oxygen (> 21%), intravenous fluids, and oral suctioning, they were more accepted to be withheld than withdrawn (Fig. 3). Concerning respiratory assistance, mechanical ventilation was more easily withheld (75%) than withdrawn (25%), just as endotracheal intubation (71–29%) and tracheostomy (67–31%).

Almost all intensivists (95%) agreed to add analgesia and or sedation to patients after the withholding/withdrawal decisions in order to prevent their suffering and allow their comfort, even though it might hasten the dying process (“Principle of Double Effect”). On another hand, 19% of the intensivists would add those therapies to actively hasten the dying process.

##### Documentation and legal aspect

Eighty-two percent of the respondents reported that they would often to always document the withholding/withdrawal decisions in the patient’s hospital record. Four percent would only sometimes do it, 6% would rarely or never trace it and 7% answered they didn’t know. When exploring the data that should be documented in the hospital record, the conversations with the family and relatives (a resume of their content) (83%) and the participants in the decision (78%) were the two most common registered information.

Sixty-one percent of the respondents found it necessary to sign a specific consent form related to the withholding/withdrawal decision, either by the patient if competent, or the surrogate or relatives if not. Moreover, 28% didn’t feel that necessity and 10% were confused about it. When asked if this consent had a legal protective value, respondents were equally positive, negative or didn’t know. Half of the intensivists reported there were no laws (54%), neither society guidelines nor recommendations controlling such practices in Lebanon (53%), nevertheless, they admitted (54%) that these practices were applied in the country. Thus, the majority of the respondents (93%) were interested to have national recommendations or guidelines for treatment limitation practices in Lebanese ICUs supported by the LNCCE and/or the Lebanese critical care societies and the LOP. Twelve percent of the intensivists had experienced a personal legal issue related to withholding/withdrawal decisions, but only one shared it in the open-ended question, stating it was limited to family anger and passed smoothly.

### Interviews

The interview with the president of LOP in Beirut clarified that the withholding/withdrawal concepts were known in Lebanon and practiced, despite not following specific guidelines or recommendations. The lawyer at the LOP agreed with that. He stated the law No. Two hundred eighty-eight of the Lebanese Medical Ethics Law of 2004, updated in February 2012/Chapter2/Article27–11 [31], covers this subject, though, vaguely. Nevertheless, it was clear enough to condemn active euthanasia and promoted a multidisciplinary approach, involving necessarily the family, but with no specifications related to the ICU, concerning the different therapies or the way of withholding or withdrawing them. He clarified, from a legal point of view, that the withholding/withdrawal decisions once discussed collegially and approved with the patient’s relatives, must be documented in the patient’s hospital record; this included a resume of the conversation, the participants in the conversations, the decision itself, with the therapies to be withheld or withdrawn. A specific consent form must then be signed by the family representatives and the doctors accordingly. The latter holds a legal protective value. The presidents of the scientific critical care societies (LSCCM: Lebanese Society of Critical Care Medicine and LSA: Lebanese Society of Anesthesiologists) explained how difficult it is to inventory the opinions of the intensivists in the country especially their practices, and much more, to unify and implement national guidelines for those decisions, because of the diversity of thoughts, curriculums, cultures and religious beliefs. The Lebanese National Consultative Committee on Ethics (LNCCE), represented by its vice-president, declared that it helped implementing palliative care in Lebanon, in various hospital units, since 1995 despite all local restraints. Lately, it is working more on this concept in the ICU by actively working on spreading the idea of patients’ “advance directives” and recently elaborating recommendations for withholding/withdrawal of life-sustaining treatments in ICUs.

The interviews with the religious leaders highlighted the diversity of their opinions regarding this subject. On the Christian side, the Catholic Church allows the withholding/withdrawal practices and has supporting detailed recommendations [32, 33], whereas the Orthodox Church allows those practices as well, but does not have clear published directives on the subject; it treats more on a case-by-case basis. For the Muslim Sunni, high authority directives (*Saudi Ulama Fatwa*) exist since 1981 [article 62 of the Islamic Code of Medical Ethics (Code of Conduct 1981)]. They allow withholding/withdrawal of life support in ICU and detail several specific situations. The only necessary and essential condition for that is the professional opinion of three specialized, reliable medical doctors [34]. All Christians and Muslim Sunni agreed with the “Principle of Double Effect”*.* For the Muslim Shia, all the main religious references (*marjaa*) stood against the withholding and withdrawal of LSTs, in all its forms and stages, no matter what its triggers were. They allowed the addition of analgesics and sedative to comfort the patients and ease their suffering only if such medications did not hasten patient’s death. Finally, the Druze form a monotheistic religion specific to the Middle East region. As Shia, they have personal “*Ijtihad*” of religious notorieties, dealing with ethical subjects. Hence, they did not have a unified position concerning the withholding/withdrawal of life-sustaining treatments in the ICU. Some of the religious figures agreed with the Sunni directives, but gave more place to the family in these decisions. Others may categorically condemn such practices, because God Almighty gave life and only He can decide when to take it back, and because of specific considerations about the soul. As for the “Principle of Double Effect”, it was preferable to avoid it as long as the patient can bear. Table 2 lists the various religious affiliations and their positions on euthanasia, withholding and withdrawing life-sustaining treatments and the principle of double effect.

**Table 2:** The different religious leaders’s affiliations and their positions on euthanasia, withholding and withdrawing (Wh/Wd) life-sustaining treatments and the principle of double effect

Religious Leader		Position	
	**Euthanasia**	**Wh/Wd**	“Principle of Double Effect”
Catholic	No	Yes Guidelines exist	Yes*
Orthodox	No	Yes No Guidelines	Yes*
Sunni	No	Yes Guidelines exist	Yes*
Shia	No	No	Yes**
Druze	No	No clear position	Preferably No only if unbearable pain

^*^ “Principle of Double Effect”: alleviation of pain is allowed, even if it *unintentionally* hastens death;

^**^ Alleviation of pain is allowed, if it will in no way lead to the patient’s death;

## Discussion

This is one of a few studies of a poorly explored topic in the Middle East: the attitudes, practices and opinions towards the withholding/withdrawal of life-sustaining treatments in ICUs in Lebanon. Our cross-sectional, qualitative study confirmed that withholding and withdrawal of life-sustaining treatments in the ICU is a familiar concept in Lebanon, consistent with most Western countries, but controverted in many Asian countries [16, 25, 35–37].

In our study, almost all intensivists perceived an ethical difference between withholding and withdrawal of life-sustaining treatments, 75% found more difficulty in withdrawing than withholding treatments, which is consistent with the previous findings from Lebanon [10].

The reasons for the withdrawal and withholding decisions in our study did not differ from those reported in most studies in the literature [10, 16, 17, 20, 22, 38]. Futility of care and poor prognosis for the chronic disease were the most common reasons cited (57%). The patient’s future quality of life accounted for 37%, and was among the main four considered reasons. Thus, Lebanese intensivists did and still agree ethically against medical treatment’s aggressiveness in ICU. The patient’s wishes were considered by 34% of intensivists, a new interesting perspective of implication of the patient that wasn’t previously taken into consideration in the Lebanese study of 2005. Economic issues and the lack of resources (0%) weren’t taken into consideration at all by Lebanese intensivists, in line with Western studies [17, 22] and a previous Lebanese study [10]. This result was obtained even though the Lebanese healthcare system was and is still economically very limited and it is conceivable that a lack of resources may lead to more withholding and withdrawal of life-sustaining treatments. However, this is usually not the case as demonstrated by Phua et al. [36] and Lobo et al. [23], probably because of a lower public trust in the health care systems of low- to middle-income economies [36].

Our study was interesting in the fact that it evaluated the tendency for every specific therapy to be withheld or withdrawn, in agreement with two French studies [16, 22]. The withheld therapies were relatively equivalent, with predominance to limit the vasopressors and inotropes, the cardiopulmonary resuscitation, the mechanical ventilation and endotracheal intubation, the hemodialysis and surgeries. However, concerning the withdrawn treatments, mechanical ventilation with terminal extubation was easier for intensivists in France than in Lebanon. Several factors may influence this practice, such as physician’s beliefs and opinions, and local policies [2]. In Lebanon, the withdrawal of mechanical ventilation once installed is illegal, except for the brain-dead patient. Our results were close to those of Yazigi et al. [10], and consistent with recent findings from a survey among Italian anesthesiologists [2], and the two studies of Phua et al. among Asian countries [36, 39], where mechanical ventilation, nutrition, fluid therapy and supplemental oxygen were usually continued.

Our study showed that the intensivists’ vision regarding the “Principle of Double Effect” has matured. Indeed, 95% of them agreed on it in our survey. This is majorly due to the tremendous efforts of the LNCCE to implement palliative care in the Lebanese health system since 1995, the easier access by specialized professionals to narcotics and the medical and public awareness drawn over the follow-up of patients after treatments limitation. In addition, the updated Medical Ethics Law No.288 [Chapter 1, Article 27–11] in February 2012, advocates soothing patients’ suffering [31]. Thus, intensivists working in Lebanon are now more locally familiar with this concept, in addition to the knowledge they are gaining in abroad trainings. This makes the post-decision follow-up in Lebanon more compatible with international guidelines [2, 22, 36, 40].

The vast majority of the Lebanese intensivists (92%) agreed that withholding and withdrawal decisions should be often to always multidisciplinary. This is in accordance with the most recent update of the Lebanese Medical Ethics Law No.288 [Chapter 1, Article 27–11] [31].

The documentation of withholding and withdrawal decisions in the patient’s hospital records is considered a sensitive part of limitation practices, because of the legal insecurity and fear it can generate. In our survey, the majority of the intensivists (82%) claimed to document their limitation decisions, most of the time (i.e. often to always). This finding resembles that of our Lebanese colleagues where only 23% of the decisions were not noted in the patient’s medical record, probably because of the old Lebanese legal context [10]. Similarly, a European study by Vincent et al. found that because of legal concerns physicians in Italy, Spain, and Greece were willing to give verbal orders to restrict care but not to write them down [25]. Even recently, this lack of documentation is still a striking concern in Norway despite the presence of a Norwegian law and clear national guidelines [13, 17]. Hence, even with the presence of laws and professional guidelines, documentation remains an important issue.

A novel aspect in our study is the assessment of religious beliefs and their influence over the withholding/withdrawal decisions. Our Lebanese colleagues did not explore it in their paper [10].. Literature showed that the religious affiliation of both physicians and patients markedly influences the withholding/withdrawal decisions in the ICU [28, 29, 36, 40, 41]. Religious beliefs can easily lead to clashes, but physicians can help prevent these conflicts by becoming knowledgeable and respecting their patients’ faiths and beliefs. This is particularly relevant in Lebanon, where many religions and their confessions are tightly intricate. Our interviews showed that not all religions have distinct rulings on the withholding/withdrawal issues, but pointed out that such rulings have to be developed.

### Limitations and strengths

This study has some limitations. First, this was a cross-sectional study and not a clinical prospective one. It assessed intensivists’ attitudes using a survey rather than recorded actual clinical practice in ICUs. An inherent limitation of such surveys is that reliability of individual responses cannot be ensured since there is always a gap between real life and declaration in response to a questionnaire. Comparison with results from clinical works is not always accurate. Future studies should be conducted, with their results confirmed by an audit. Second, the sample size recruited was relatively small, even after specific regrouping of variables. Finally, withholding and withdrawal were considered as one aspect; separate questions for withholding and withdrawal are important to consider in future studies since they are not the same, even if experts in ethics consider them similar.

The strengths of our study resided in its novelty and its qualitative aspect, which provided depth that a cross-sectional survey could not possibly provide. It assessed a poorly explored subject in Lebanon, on a wide scale. It also combined an intensivists’ survey with leaders’ opinion interviews. Moreover, it shed the light on an ethno-religious group, specific to the Middle East region, the Druze religion, which to our knowledge, was not previously studied in international papers. The study also showed that the rights to dispose of one’s health conferred on citizens by law (advance directives, surrogate) became more familiar to the intensivists in Lebanon.

## Conclusion

This study revealed that Lebanese intensivists’ practices are not far away from what is recommended and applied internationally. Cultural attitudes are not easily changed, however this study showed the effective evolution of thoughts and attitudes toward the limitation of treatment in Lebanese ICUs, which accompanied the modern evolution of the ethico-legal framework in the country. This study has potentially important implications for the withholding/withdrawal of life-sustaining treatments in ICUs in Lebanon. Along with the rapid expansion of availability of ICU services, the ethical dimension of limiting treatments requires greater discussion within the health care community and the public. Our data provides an opportunity to spread awareness over this subject among the medical community, as a start and allows a comparison of the previous and current Lebanese practices and the situation of the actual ones among Western and Eastern countries. The context and characteristics of decisions to withhold or withdraw therapies in Lebanon do not differ greatly from the Western countries. Further investigations should analyze specific aspects of the withholding/withdrawal decisions in details, study physicians’ practical attitudes and the perceptions of patients and families, in order to elaborate clear, national and relevant guidelines and optimize these decisions in Lebanese ICUs.

## Supplementary information

**Additional file 1.** Appendix 1. This file includes the questionnaire used for this study.

**Additional file 2.** Appendix 2. This file includes the original 5-point Likert scale table before merging of categories and after merging the “Rarely” and “Never” categories.

## Data Availability

All data generated or analyzed during this study are not publicly available to maintain the privacy of the individuals’ identities. The dataset supporting the conclusions is available upon request to the corresponding author.

## Abbreviations

ICU: Intensive Care Unit
WFSICCM: World Federation of Societies of Intensive and Critical Care Medicine
LSA: Lebanese Society of Anesthesiologists
LSCCM: Lebanese Society of Critical Care Medicine
LOP: Lebanese Order of Physicians
LNCCE: Lebanese National Consultative Committee on Ethics
CPR: Cardio-Pulmonary Resuscitation
ECMO: Extracorporal Membrane Oxygenation
LST: Life-sustained treamtent
