# Supplementary material for: Withholding and withdrawal of life-sustaining treatments in intensive care units in Lebanon: a cross-sectional survey of intensivists and interviews of professional societies, legal and religious leaders
Source: BMC Med Ethics. 2020 Aug 28;21:80. doi: 10.1186/s12910-020-00525-y (PMC7456082; doi:10.1186/s12910-020-00525-y)
Supplement: Supplementary file 1 — Additional file 1. Appendix 1. This file includes the questionnaire used for this study. [file 12910_2020_525_MOESM1_ESM.docx]

Appendix 1

**1. Background information**

**1.1. Gender:** □ Female □ Male

**1.2. Age:** □ < 30 years

□ > 30 years - < 39 years

□ > 40 years - < 49 years

□ > 50 years - < 59 years

□ > 60 years

**1.3. Religion:** □ None

□ Christian: □Catholic □Orthodox □Protestant

□ Muslim: □Sunni □Shia

□ Druze

□ Others __________________________

**1.4. Place of current practice:**

**1.4a. Type of Hospital :**  □ University Hospital

□ Non-University Hospital

□ Private Hospital

□ Public Hospital

**1.4b.Number of beds :**  □ < 200 beds

□ > 200 beds - < 299 beds

□ > 300 beds - < 399 beds

□ > 400 beds - < 499 beds

□ > 500 beds

**1.4c.Location of Hospital :**  □ Beirut

□ Mount Lebanon

□ North Lebanon

□ South Lebanon

□ Bekaa

**1.5. How long have you been working? (Number of years of practice)**

⁭ □ < 5 years

⁭ □ > 5 years - < 9 years

⁭ □ > 10 years - < 19 years

⁭ □ > 20 years

**1.6. You are specialized in**

⁭ □ Critical Care Medicine and Anesthesia

⁭ □ Critical Care Medicine and Pulmonary Medicine

□ Critical Care Medicine and Other medical specialty : ________________

**1.7. Did you participate in any fellowship program abroad (outside Lebanon)?**

⁭ □ Yes

⁭ □ No

⁭ □ If YES, Where (*Country*) : ________________________

**1.8. How often are you on average in the ICU?**

⁭ □ permanently

□ > 50% of my working time

⁭ □ < 50% of my working time

⁭ □ Other ________________

□ Never 🡪 Thank you for your time and effort, the rest of the questions aren’t applicable in your case

**1.9. The ICU you are affiliated with is**

⁭ □ a medical **adult** ICU

⁭ □ a surgical **adult** ICU

⁭ □ a mixed **adult** (medical and surgical) ICU

**P.S.** Pediatric ICUs are excluded, thank you for your time and effort, the rest of the questions aren’t applicable in your case

**1.10. What is the bed capacity of the ICU you are affiliated with**

⁭□ < 5 beds

⁭□ > 5 - < 9 beds

⁭□ > 10 beds

**2. Reasons for withholding/withdrawing therapy in ICU**

**2.1. Are you familiar with the concepts of Withholding and Withdrawal of life-sustaining therapy in the ICU?**

⁭ □ Yes ⁭ □ No

***Please read the following definitions before you respond***

| ***Withholding therapy*** | *Decision not to start or increase a life-sustaining intervention* |
| --- | --- |
| ***Withdrawing therapy*** | *Decision to actively stop a life-sustaining intervention presently being*  *given (with focus on cure to palliative therapy in anticipation of imminent death)* |
| ***Euthanasia*** | *Act by which the causative agent of death is administered by another with the intent to end life* |
| ***Assisted suicide*** | *Physician provides the means, patient acts* |
| ***Medical Futility*** | *A clinical action/therapy serving no useful purpose in attaining a specified goal for a given patient* |

**2.2. Do you apply Withholding and Withdrawal of life-sustaining therapy in your ICU?**

⁭ □ Yes □ No

**2.3. In your opinion, what should cause considerations about withholding or withdrawing**

**Life-sustaining therapy in the ICU?**

***Choose the 3 most important causes in your opinion and number them from 1 to 3***

⁭ □ Age

⁭ □ Poor prognosis for the acute illness/condition

⁭ □ Poor prognosis for underlying chronic disease

⁭ □ Poor prognosis for **future** quality of health and life

⁭ □ No response to (maximum) therapy / Futile therapy

⁭ □ Multiple organ failure

⁭ □ Patient’s wishes

⁭ □ Family and Relatives’ wishes

□ Psychological pain (for the patient and family)

□ Physical pain (expected suffering related to continuing ICU therapy)

⁭ □ Question of resources (space problems, ICU bed shortage)

⁭ □ Previous patient’s physical condition (autonomy)

⁭ □ Previous patient’s mental condition

□ Economical issues (high cost of hospitalisation, low family income, financial burden for family and institution)

□ Other ______________________

**2.4a. Is there for you, an ethical difference between withholding therapy and withdrawing therapy?**

⁭ □ Yes □ No □ Don’t know

**2.4b. If yes; what is the most difficult to make decisions about?**

□ Withhold therapy □ Withdraw therapy

**And WHY ?** __________________________________________________________________________________________________________________________________________________________________________________________________________________________________________

**3. The decision-making process in connection with withholding or**

**withdrawing of therapy at the ICU**

***Please read the following definitions before you respond***

| ***Intensivists*** | *Specialized intensive care physicians who either have their main*  *workplace in the ICU or have their main workplace elsewhere and*  *only take care of intensive care patients on shifts* |
| --- | --- |
| ***Primary treating physicians*** | *Physicians from the medical speciality the patient is initially admitted to,*  *such as abdominal surgery, neurology or internal medicine* |
| ***Surrogate decision maker*** | *Advocates who provide direction in decision-making for incompetent patients unable to make decisions or decide for themselves about personal health care* |
| ***Patient’s advance directives*** | *A document by which a person makes provision for health care decisions in the event that in the future he/she becomes unable to make those decisions* |
| ***Multidisciplinary decision*** | *Cooperatively working together, sharing responsibility for problem solving and decision-making, to formulate and carry out plans for patient care and involving all the unit intensivists, the primary physician, the ICU nurses, and the hospital ethical committee* |

**3.1. Are there, in your unit or institution, standardized protocols and/or recommendations to follow, for withholding/withdrawal decision-making?**

⁭ □ Yes □ No

**3.2. In your opinion, should the decision-making process regarding withholding or withdrawing life-sustaining therapy in the ICU :**

|  | Always | Often | Sometimes | Rarely | Never |
| --- | --- | --- | --- | --- | --- |
| Be taken with a multidisciplinary approach |  |  |  |  |  |
| Involve the patient (if competent) |  |  |  |  |  |
| Rely on the patient’s advance directives, when they exist (if the patient is incompetent) |  |  |  |  |  |
| Involve the surrogate decision-maker, previously chosen (if the patient is incompetent) |  |  |  |  |  |
| Involve the family and relatives |  |  |  |  |  |
| Involve the primary treating physician |  |  |  |  |  |
| Involve the ICU nurses responsible of the patient |  |  |  |  |  |
| Involve the hospital ethical committee |  |  |  |  |  |

**3.3. Do you provide clear and complete information about the patient’s medical status to the family/relatives or surrogate prior to the decision-making process in your unit?**

□ Always

⁭ □ Often

⁭ □ Sometimes

⁭ □ Rarely

⁭ □ Never

**4. Practices of withholding or withdrawing of therapy in the ICU**

**4.1.What therapies do you think should be withheld and/or withdrawn?**

*(You can tick off more than one answer)*

| **TREATMENTS** | **WITHHELD** | **WITHDRAWN** |
| --- | --- | --- |
| Cardiopulmonary resuscitation |  |  |
| Vasopressors / Inotropes |  |  |
| Endotracheal intubation |  |  |
| Tracheotomy |  |  |
| Mechanical ventilation |  |  |
| Hemodialysis |  |  |
| Broad-spectrum antibiotics |  |  |
| Blood products transfusion |  |  |
| Nutrition : enteral and parenteral |  |  |
| Anticoagulation |  |  |
| Diuretics |  |  |
| Oral suctioning |  |  |
| Intravenous fluid therapy |  |  |
| Supplemental Oxygen > 21% |  |  |
| Further surgeries (urgent or not) |  |  |
| Non-invasive ventilation |  |  |
| ECMO |  |  |
| Total therapies |  |  |

**4.2. In your opinion, after withholding/withdrawal decisions,**

|  | **YES** | **NO** |
| --- | --- | --- |
| **should analgesia and/or sedation be added to prevent patients’ suffering?** |  |  |
| **should analgesia and/or sedation be added to allow patients’ comfort?** |  |  |
| **should analgesia and/or sedation be added to actively shorten the dying process?** |  |  |
| **analgesia and sedation shouldn’t be added to avoid accelerating the patient’s death (despite pain, suffering and discomfort)?** |  |  |

**5. Documentation and Legal aspect**

**5.1. In your opinion, should the withholding/withdrawal decisions be recorded in the**

**patient’s hospital record?**

□ Always

⁭ □ Often

⁭ □ Sometimes

⁭ □ Rarely

⁭ □ Never (tacit decision)

**5.2. What should in your opinion be documented in the hospital records regarding**

**withholding or withdrawing therapy?**

*(You can tick off more than one answer)*

⁭ □ Conversations with family and relatives (a resume of content)

⁭ □ Conversations with the patient when competent (a resume of content)

⁭ □ Participants in the conversations

⁭ □ Participants in the decision of withholding/withdrawal of therapies

⁭ □ Background for decisions (reasons)

⁭ □ The decision itself regarding withholding or withdrawing therapy

⁭ □ What specific therapy would be withheld or withdrawn

⁭ □ How therapy should be terminated (progressively, changing doses, molecules…)

□ Other ______________________

**5.3. In your opinion, once the withholding/withdrawal decisions are taken, should the patient (if competent), or the surrogate, or the relatives sign a consent form of their decision?**

⁭ □ Yes □ No □ Don’t know

**5.4. In your opinion, does this consent form have a legal protective value?**

⁭ □ Yes □ No □ Don’t know

**5.5. In your knowledge, are there any laws in Lebanon allowing and controlling these practices of withholding and withdrawal of life-sustaining therapies in the ICU?**

⁭ □ Yes □ No □ Don’t know

**5.6. In your knowledge, are there in Lebanon society guidelines and recommendations concerning the withholding and withdrawal of life-sustaining therapies in the ICU?**

⁭ □ Yes □ No □ Don’t know

**5.7. In your knowledge, are these decisions of withholding and withdrawal of life-sustaining therapies in the ICU practiced in Lebanon?**

⁭ □ Yes □ No □ Don’t know

**5.8. In your personal experience, have you ever had legal issues related to the withholding/withdrawal of life-sustaining therapies decisions in the ICU?** ⁭

⁭ □ Yes □ No

*Share a personal experience*  __________________________________________________________________________________________________________________________________________________________________________________________________________________________________________

**5.9. Would you like to have national recommendations or guidelines of end of life practices in the ICU supported by the Lebanese National Consultative Committee on Ethics (LNCCE) and the Lebanese critical societies (LSA and LSCCM)?**

⁭ □ Yes □ No
