# Supplementary material for: Withholding and withdrawal of life-sustaining treatments in intensive care units in Lebanon: a cross-sectional survey of intensivists and interviews of professional societies, legal and religious leaders
Source: BMC Med Ethics. 2020 Aug 28;21:80. doi: 10.1186/s12910-020-00525-y (PMC7456082; doi:10.1186/s12910-020-00525-y)
Supplement: Supplementary file 2 — Additional file 2. Appendix 2. This file includes the original 5-point Likert scale table before merging of categories and after merging the “Rarely” and “Never” categories. [file 12910_2020_525_MOESM2_ESM.docx]

Appendix 2

| Supplementary Table 1. Descriptive data showing the answers of the participants according to six-option categories. | | | | | | |
| --- | --- | --- | --- | --- | --- | --- |
|  | Always | Often | Sometimes | Rarely | Never | Don't Know |
| In your opinion, should the decision-making process regarding withholding or withdrawing life-sustaining therapy in the ICU |  |  |  |  |  |  |
| Be taken with a multidisciplinary approach | 59 (71%) | 17 (21%) | 5 (6%) | 0 (0%) | 2 (2%) |  |
| Involve the patient (if competent) | 42 (51%) | 12 (14%) | 17 (21%) | 5 (6%) | 7 (8%) |  |
| Rely on the patients' advance directives when they exist (if the patient is incompetent) | 41 (49%) | 23 (28%) | 11 (13%) | 4 (5%) | 4 (5%) |  |
| Involve the surrogate decision-maker previously chosen (if the patient is incompetent) | 42 (51%) | 25 (30%) | 11 (13%) | 3 (4%) | 2 (2%) |  |
| Involve the family and relatives | 28 (33%) | 29 (35%) | 18 (22%) | 5 (6%) | 3 (4%) |  |
| Involve the primary treating physician | 45 (54%) | 33 (40%) | 4 (5%) | 0 (0%) | 1 (1%) |  |
| Involve the ICU nurses responsible of the patient | 35 (42%) | 30 (36%) | 9 (11%) | 8 (10%) | 1 (1%) |  |
| Involve the hospital ethical committee | 40 (48%) | 22 (27%) | 15 (18%) | 5 (6%) | 1 (1%) |  |
| Do you provide clear and complete information about the patient's medical status to the family/relatives or surrogate prior to the decision-making process in your unit? | 63 (76%) | 18 (22%) | 2 (2%) | 0 (0%) | 0 (0%) |  |
| In your opinion, should the withholding/withdrawal decisions be recorded in the patient's hospital record? | 54 (65%) | 14 (17%) | 3 (4%) | 1 (1%) | 4 (5%) | 6 (8%) |

Different *n* due to missing data); Percentages may not total 100 because of rounding)

| Supplementary Table 2. Descriptive data showing the answers of the participants according to five-option categories. | | | | | |
| --- | --- | --- | --- | --- | --- |
|  | Always | Often | Sometimes | Rarely and Never | Don't Know |
| In your opinion, should the decision-making process regarding withholding or withdrawing life-sustaining therapy in the ICU |  |  |  |  |  |
| Be taken with a multidisciplinary approach | 59 (71%) | 17 (21%) | 5 (6%) | 2 (2%) |  |
| Involve the patient (if competent) | 42 (51%) | 12 (14%) | 17 (21%) | 12 (14%) |  |
| Rely on the patients' advance directives when they exist (if the patient is incompetent) | 41 (49%) | 23 (28%) | 11 (13%) | 8 (10%) |  |
| Involve the surrogate decision-maker previously chosen (if the patient is incompetent) | 42 (51%) | 25 (30%) | 11 (13%) | 5 (6%) |  |
| Involve the family and relatives | 28 (33%) | 29 (35%) | 18 (22%) | 8 (10%) |  |
| Involve the primary treating physician | 45 (54%) | 33 (40%) | 4 (5%) | 1 (1%) |  |
| Involve the ICU nurses responsible of the patient | 35 (42%) | 30 (36%) | 9 (11%) | 8 (11%) |  |
| Involve the hospital ethical committee | 40 (48%) | 22 (27%) | 15 (18%) | 6 (7%) |  |
| Do you provide clear and complete information about the patient's medical status to the family/relatives or surrogate prior to the decision-making process in your unit? | 63 (76%) | 18 (22%) | 2 (2%) | 0 (0%) |  |
| In your opinion, should the withholding/withdrawal decisions be recorded in the patient's hospital record? | 54 (65%) | 14 (17%) | 3 (4%) | 5 (6%) | 6 (8%) |
